# A Case of Coexisting Depression and Hoarding Disorder

**DOI:** 10.7759/cureus.25645

**Published:** 2022-06-03

**Authors:** Neeraj Kancherla, Sameer Krishna Prasad Garlapati, Panakala Sowkhya, Tejaswini Yegateela, Amanda McCrary

**Affiliations:** 1 Psychiatry, King George Hospital, Visakhapatnam, IND; 2 Internal Medicine, Andhra Medical College, Visakhapatnam, IND; 3 Internal Medicine, King George Hospital, Visakhapatnam, IND; 4 Psychiatry, Andhra Medical College, Visakhapatnam, IND; 5 Psychiatry and Behavioral Sciences, Pontiac General Hospital, Pontiac, USA; 6 Psychiatry, University of Medicine and Health Sciences, Basseterre, USA

**Keywords:** obsessive-compulsive disorder, hoarding disorder, psychiatry, depression, mental health

## Abstract

A hoarding disorder manifests as difficulty in discarding or letting go of items irrespective of their actual worth and persistent acquisition of items. Increasing numbers of possessions clutter active living spaces to the point where their intended use is no longer possible, leading to significant functional impairment. In the Diagnostic and Statistical Manual of Mental Disorders (DSM-V), it is classified under the category obsessive-compulsive and related disorders. First considered to be a part of obsessive-compulsive disorder, compulsive hoarding was subsequently classified as an additional dimension of the obsessive-compulsive disorder spectrum. Hoarding disorder had been largely ignored clinically until recently, despite negative consequences on individuals, families, and communities. Comorbid conditions like anxiety, depression, obsessive-compulsive disorder, post-traumatic stress disorder, and panic disorder are well known to accompany hoarding disorder. This study illustrates the case of a 35-year-old married man who was referred to a psychiatrist by his primary care physician for collecting many different objects of little or no importance. These objects were lying unorganized throughout his house and cluttering most of his living space. Some of these things were discarded by his wife, which, according to him, contributed to his emotional distress. The pattern of behavior began about 10 years earlier, and it became increasingly problematic with time. Although hoarding disorder is often underreported, it is vital to diagnose this condition as it significantly affects the individual and their family and friends. Severe hoarding can pose a number of health and safety risks, including fire hazards, tripping hazards, and health code violations.

## Introduction

In the Diagnostic and Statistical Manual of Mental Disorders (DSM-V), the diagnosis of hoarding disorder (HD) was added as an independent diagnosis, replacing its inclusion in the DSM-IV as an obsessive-compulsive disorder. The hoarding condition may lead to impairment in such basic functions as eating, sleeping, and grooming [1]. HD is characterized by a persistent inability to discard items, the desire to save items instead of discarding them, significant accumulations of possessions that clutter living spaces, and significant dysfunctional patterns. The items are typically considered to be useful in the future, aesthetically impressive, or have an emotional connection [1]. The prevalence of hoarding disorder in the general population is estimated at 2.6%, with higher rates among people over 60 years of age and individuals with other psychiatric diagnoses [2]. Compared to those in the general population, individuals with hoarding disorder tend to be older, unemployed, and unmarried, separated, or divorced more often [3]. A majority of hoarders do not consider their behavior problematic. Typically, hoarding patients accumulate items passively rather than intentionally, which leads to a gradual accumulation of clutter over time. Those with HD may experience distress similar to those with obsessive-compulsive disorder (OCD) under similar circumstances when their possessions are touched or moved without their permission. The two conditions, however, differ significantly. OCD is characterized by obsessive or intrusive thoughts which are repetitive and unwanted, while in HD, thoughts regarding the keeping or acquisition of objects are not considered intrusive or unwanted. While intrusive thoughts are distressing in OCD, distress in HD results more from the consequences of thoughts or behaviors than from the thoughts or behaviors themselves [4]. As HD progresses through each decade of life, symptoms deteriorate, and the level of distress would increase if the family or authorities intervened. In many cases, HD occurs in conjunction with other psychiatric disorders. Major depression is the most common comorbidity, occurring in as many as 50% of cases [5]. 

The anterior cingulate cortex (ACC) and related areas of the brain appear to be particularly associated with hoarding. The ACC in the dorsal hemisphere is involved with decision-making and reward-based learning, while the ACC in the ventral hemisphere is associated with emotional and motivational experiences. Observations of functional magnetic resonance imaging studies have shown that ACC activation is lower in hoarding individuals compared to control individuals [6,7]. Usually, hoarding disorder is diagnosed based on a direct interview with the person to determine whether or not the characteristics of the disorder exist. It is crucial to recognize that hoarding is not always the primary reason for consultation, as in this instance when depression and apathy were the primary reasons for consulting the primary care physician. Clinicians can direct questions using assessment scales such as the Structured Interview for Hoarding Disorder, the Clutter Image Rating, and the Hoarding Rating Scale-Interview to assess for HD [5,8,9].

## Case presentation

The patient is a 35-year-old male who was brought to his primary care physician (PCP) by his wife with complaints of lethargy and depression. His primary care physician referred him for further evaluation by a psychiatrist. The patient had no past medical history; however, his psychiatric history shows a diagnosis of generalized anxiety disorder from 15 years ago.

In the course of the initial evaluation, the patient revealed that he gets irritable and depressed when his wife discards any items in his possession. He stated that his wife had discarded more of his items over the past year, causing him to become progressively depressed. Considering his items to be valuable and likely to be useful in the future, the patient began collecting mail, magazines, tissue rolls, clothing, cooking supplies, and snacks. He has the habit of purchasing in greater quantities than he needs in the expectation that his purchases will prove useful in the future. Despite never using these items, he could not make himself get rid of them. He acknowledges that his habit of excessive acquisition and behavior regarding difficulties in discarding these items is problematic and troubling for his wife as well. While he could not discard his possessions because of their possible use, he did not consider his thoughts about them to be repetitive or distressing, despite the fact the possessions were taking over his living areas. There were objects piled up in every room in his house, including his living room, bedroom, basement, garage, and surrounding areas. 

The patient has no psychiatric history in the family. A physical examination showed no abnormalities. Manic or psychotic symptoms are denied by the patient. The patient also denied having suicidal thoughts. The patient had no history of alcohol or substance abuse.

A mental status evaluation revealed a well-groomed middle-aged man. The patient was alert, aware, and oriented toward his surroundings. His behavior was cooperative and calm throughout the interview, and he made appropriate eye contact. He interacted in a rational manner throughout. His speech was organized and coherent. He described his mood as depressed, and he had a depressed affect. In addition to fair insight, his memory, judgment, and concentration are excellent. His thought process was simple and linear. He was evaluated for hoarding disorder with the Hoarding Rating Scale, and his results were clinically significant (Table 1).

**Table 1:** Hoarding Rating Scale with patient reported scores Scoring scale: On a scale of 0-8, 0 is for no problems, 2 is mild, 4 is moderate, 6 is severe and 8 is extreme. Interpretation of HRS total scores [9] - mean for nonclinical samples: HRS total = 3.34; standard deviation = 4.97. Mean for people with hoarding problems: HRS total = 24.22; standard deviation = 5.67. Analysis of sensitivity and specificity suggest an HRS total clinical cut-off score of 14. Criteria for clinically significant hoarding [10] - a score of 4 or greater on questions 1 and 2, and a score of 4 or greater on either question 4 or question 5. HRS: Hoarding Rating Scale

1. Because of the clutter or number of possessions, how difficult is it for you to use the rooms in your home?
Score: 6
2. To what extent do you have difficulty discarding (or recycling, selling, giving away) ordinary things that other people would get rid of?
Score: 7
3. To what extent do you currently have a problem with collecting free things or buying more things than you need or can use or can afford?
Score: 7
4. To what extent do you experience emotional distress because of clutter, difficulty discarding, or problems with buying or acquiring things?
Score: 8
5. To what extent do you experience impairment in your life (daily routine, job/school, social activities, family activities, financial difficulties) because of clutter, difficulty discarding, or problems with buying or acquiring things?
Score: 6

## Discussion

In this study, we describe the case of a patient with hoarding disorder coupled with depression. A progression of accumulation behavior and inability to discard objects was observed in the patient over time. He was referred to a psychiatrist's care and diagnosed with hoarding disorder about ten years after the inception of his symptoms. The delay in visiting a physician and receiving a timely diagnosis is common with HD, resulting in worsening symptoms over time and a detrimental impact on quality of life.

In this case, the patient was experiencing emotional distress from the efforts of his wife to clean their home, and his relationship with her was suffering. The patient had good insight into the effects of his hoarding behaviors, but still found it distressing to part with items that had no usefulness to him. He attributed these resentful feelings towards his wife as the cause for his recent depressive and lethargic state. According to a number of studies, people with HD are significantly more likely to suffer from depression than people in the general population [5]. The patient has a history of generalized anxiety disorder, but no previous diagnosis of major depressive disorder (MDD). As part of the diagnosis for HD, the clinician had to rule out the symptoms of hoarding as being better explained by another psychiatric disorder. In this case, it is apparent he is not unable to throw things out due to lack of energy or a depressed state but is instead unable to throw items due to their perceived importance for a future time. As his HD progressed over the last ten years, the consequences of his disorder became more disruptive to his relationships. The patient perceives the attempts of his wife to declutter their home as contributing to his feelings of depressed mood and lethargy. 

The distress of discarding items in persons with HD is associated with functional changes in the brain in areas regulating anxiety and sadness when compared to controls [11]. Although the precise cause of hoarding disorder is unknown, this statistically significant difference may help explain the distress caused to the patient by his wife's efforts. The lasting effects of his sadness, including behavioral changes not previously seen before and psychomotor retardation, are related to his hoarding disorder but meet the criteria for an independent diagnosis of major depressive disorder. This case illustrates one example of comorbid mood disorder with hoarding disorder. The primary treatment for hoarding disorder is cognitive behavioral therapy (CBT), which includes psychoeducation, motivational interviewing, classic cognitive procedures centered on dysfunctional beliefs, and exposures focusing on sorting and discarding. Some pharmacological therapies could also benefit patients. This patient was scheduled to participate in cognitive behavioral therapy sessions on a weekly basis in order to gain an understanding of the patient's beliefs, educate him, and make him aware of the importance of treatment. The sessions also focused on the cognitive restructuring of his beliefs to curb his compulsive purchasing and guide him on discarding objects from his house. In addition to CBT, fluoxetine 10mg, once daily, is prescribed to manage his depression symptoms. A family therapy session has been suggested for him and his wife. He had a monthly appointment with the psychiatrist to monitor his progress. There was no notable improvement throughout the initial three months of treatment. However, by the fourth and fifth months, the patient had stopped compulsive purchasing. By the end of ten months, he could relieve his house of unnecessary possessions. His depression symptoms had returned to normal levels, and he reported feeling happy and optimistic.

## Conclusions

Hoarding disorder is characterized by a persistent inability to discard or part with possessions, irrespective of their value. They see a need to preserve the items and experience discomfort when confronted with the possibility of discarding them. It is critical to understand that hoarding is not often the major cause for consultation, as was the scenario in this case report, where depression and lethargy were the key reasons for consulting the primary care physician. Overbuying and hoarding multiple items throughout his house has strained his relationship with his wife due to his unwillingness to discard them. Attempts to discard the items from the house have led to depression in our patient. Hence, it is important to recognize that patients with hoarding disorder may also suffer from anxiety or depression that needs to be treated as well.

Hoarding can severely impact the lives of affected patients and those around them if it is not diagnosed and treated early. In addition to CBT, interpersonal and family therapy should be considered in order to further improve the patient's relationships with family and friends. There is a need for studies evaluating treatments for HD in order to better the quality of life of the patient and to reduce the possible hazards associated with the disorder.
